# Astrocytes As the Main Players in Primary Degenerative Disorders of the Human Central Nervous System

**DOI:** 10.3389/fnagi.2016.00045

**Published:** 2016-03-04

**Authors:** Francisco Capani, Cecilia Quarracino, Roberto Caccuri, Roberto E. P. Sica

**Affiliations:** ^1^Instituto de Investigaciones Cardiologicas ININCA UBA CONICETBuenos Aires, Argentina; ^2^Instituto de Ciencias Biomédicas, Facultad de Ciencias de la Salud, Universidad Autónoma de ChileTemuco, Chile

**Keywords:** astrocytes, primary degenerative disorders, human central nervous system, neurodegeneration, primary astrocytic degeneration

## Abstract

Along the last years it has been demonstrated that non-neural cells play a major role in the pathogenesis of the primary degenerative disorders (PDDs) of the human central nervous system. Among them, astrocytes coordinate and participate in many different and complex metabolic processes, in close interaction with neurons. Moreover, increasing experimental evidence hints an early astrocytic dysfunction in these diseases. In this mini review we summarize the astrocytic behavior in PDDs, with special consideration to the experimental observations where astrocytic pathology precedes the development of neuronal dysfunction. We also suggest a different approach that could be consider in human investigations in Alzheimer’s and Parkinson’s disease. We believe that the study of PDDs with human brain samples may hold the key of a paradigmatic physiopathological process in which astrocytes might be the main players.

## First Steps: Neurodegeneration

Since the beginning of the 20th century relentlessly progressive neurological diseases have classically been attributed to a primary neuronal dysfunction. This affirmation applies to Parkinson disease (PD), Alzheimer disease (AD), amyotrophic lateral sclerosis (ALS) and frontotemporal dementia (FTD), among others (Pendlebury et al., 1987; Manetto et al., 1988). Therefore, most investigations have been carried out under this belief, leading to the coining of the term “neurodegeneration.” This paradigm was later on applied to psychiatric diseases such as Schizophrenia and Major depression (Harrison, 1999; Delgado, 2004). As a result of those efforts, a detailed description of neurons’ characteristics in the main affected areas in those diseases as well as a broadening of the knowledge of neuronal functioning was achieved. Since then, several physiopathological hypotheses were constructed; notwithstanding that when put into practice by therapeutical testing, results have not met the expectations (McDermott and Rowland, 2001; Broadstock et al., 2014).

In the 90s, a new era began that considered the possibility of a more important role of the so far neglected neuroglia. This led to different findings opposed to the belief of the astrocytes sole function being neuronal structural support.

Astrocytes have been found to have a much more active role that the one predicted by the earlier guessers. Particularly, they are involved in the ions exchange with neurons, they are organized as a syncytium that allows them to interchange information with other astrocytes residing in a defined net through different types of Ca^+++^ signals while regulating the release of signaling molecules involved in the production of trophic factors, transmitters and transporters that when released to the extracellular medium will modulate the synaptic activity synchronizing the neuronal functions. Also they are involved in the extracellular K^+^ uptake, in synaptogenesis and gene expression; adapting, at the same time, the permeability of the blood–brain barrier to the neuronal and synaptic needs (Reichenbach et al., 2010; Gordon et al., 2011; Rash et al., 2012; Khakh and Sofroniew, 2015). A more exhaustive description of the astrocytes properties and functions is beyond the scope of this mini review and is detailed elsewhere (Sica, 2015).

## The New Era: Primary Degenerative Disorders

Slowly but relentlessly, the results of several studies confirmed the existence of an active role played not only by the astrocytes but also by microglia. Mounting evidence suggests that astrocytes modulate microglial response, through the establishment of a complex cross-talk between both types of cells mediated by the production of different chemokines and cytokines (Cudaback et al., 2015; Rothhammer and Quintana, 2015). Therefore, we think that the broader term primary degenerative disorders of the central nervous system (PDD CNS) alludes to the complex pathology of these diseases (in contrast to the classic term neurodegeneration) and will be used henceforward in this article.

Astrocytic findings in the PDDs include:

In FTD, the classic approach subdivided the frontotemporal anatomic atrophy with regards to the concomitant histopathological findings, that is: Pick disease when tau-positive intraneuronal inclusions were found, FTD with motor neuron disease when tau-negative-ubiquitine-positive intracellular inclusions were present in motor neurons or Frontotemporal Lobar Degeneration when no particular histopathological features were observed (Broe et al., 2004). On the contrary, recent studies have demonstrated that regardless of the presence or absence of the “classic” neuronal inclusions, clinical disease progression is associated with the extent of neuronal loss, the degree of extracellular microvacuolation and, regarding glial pathology, the level of both astrogliosis and astrocytic apoptosis (Broe et al., 2004; Kersaitis et al., 2004).

With regards to PD, microgliosis has been found in a sustained fashion by different authors associated with the creation and maintenance of a vigorous inflammatory state at the substantia nigra pars compacta (McGeer and McGeer, 2008; McGann et al., 2012) and striatum, the latter of a strikingly similar characteristics in animal models and human brain samples (Charron et al., 2014). The mentioned state seems to be modulated by astrocytes, through the exocytosis of paracrine factors in the substantia nigra such as ICAM-1 and IL-6, which in turn act as stimulators of microglia migration (McGeer and McGeer, 2008).

Regarding AD, in a triple transgenic mouse model it has been shown that the presence of senile plaques in the hippocampus induces a mild astrocytic (and microglial) reaction within the Aβ plaques’ periphery (Olabarria et al., 2010); a finding that was not observed in the entorhinal or prefrontal cortex (Yeh et al., 2011; Kulijewicz-Nawrot et al., 2012). However, in human brain samples astroglial reaction, although present and with a clear hippocampal preference, seems to be less clearly associated with amyloid deposition at least in the lateral temporal cortex (Wharton et al., 2009; Olabarria et al., 2010; Simpson et al., 2010; Verkhratsky et al., 2010, 2014a; Yeh et al., 2011; Prokop et al., 2013).

Both in Major Depression and Schizophrenia there seems to be a mild astrocytic atrophy (characterized mainly by a decreased expression of GFAP) without a definitive consensus in relation to the stability of the total number of astrocytes (Verkhratsky et al., 2014b; Verkhratsky and Parpura, 2015). Moreover, in Schizoprhenia, several functional astrocytic changes have been described such as a diminished expression of EAAT1 and EAAT1 (human glutamate transporters) in the prefrontal cortex, augmented synthesis of condroitin sulfate proteoglycans in the amygdala and entorhinal cortex, altered metabolism of D-serine, elevated levels of kynurenic acid (NMDA inhibitor synthesized by astrocytes), etc. (Rajkowska and Miguel-Hidalgo, 2007; Alexander et al., 2012; Feresten et al., 2013; Verkhratsky et al., 2014b; Verkhratsky and Parpura, 2015).

Finally, in ALS, astrogliosis both in white and gray matter is a very important hallmark of the illness all along its course (Yamanaka et al., 2008; Lasiene and Yamanaka, 2011; Sica, 2012). In the SOD1^G93A^ transgenic mouse model astroglial atrophy has been described restricted to the astrocytes associated with the affected motor neurons (Rossi et al., 2008). Microglia activation is also increased in ALS and in another SOD1 mouse model Yamanaka et al. (2008) demonstrated that it could be modulated by the manipulation of astrocytic behavior (Yamanaka et al., 2008; Lasiene and Yamanaka, 2011; Sica, 2012).

### Special Consideration

A particular difficulty in the study of these diseases is that they mostly represent a deterioration of a highly evolved cognitive and/or motor functioning system. Therefore, and punctually in psychiatric illnesses, it seems that animal models are far from resembling all the peculiarities of the human diseases, even if the anatomopathological findings can be mimicked. On the other hand, human testing is ethically inappropriate for obvious reasons and therefore, the most representative research tool available is the study of post mortem human brains (Harrison, 1999; Broadstock et al., 2014). Furthermore, since clinical diagnosis relies in a constellation of signs and symptoms that are secondary to a physiopathogenic process that is far from recently starting, the study of the disease early-stages is extremely difficult. All the above determines hazards to the methodological study approach of many hypotheses, particularly those involving disease onset and progression.

## Temporality Clues

We reviewed the international literature in order to find descriptions in human or animal models which may support the hypothesis that astrocyte pathology precedes neuronal damage (**Table 1**).

**Table 1:** Experimental findings suggestive of primary astrocytic damage.

Disease	Reference	Species	Findings
FTD	Broe et al., 2004	Human	Astrocytic apoptosis is present at early stages and increases with disease progression.
	Kersaitis et al., 2004	Human	Astrogliosis is present at early stages of the disease.
PD	Halliday and Stevens, 2011	Mice	Astrocytic cytoplasmatic α-synuclein deposition precedes neuronal damage.
AD	Olabarria et al., 2010	Mice (3xTg)	Hippocampal astroglia atrophy (6th month) precedes the extracellular Aβ deposition (12th month).
	Yeh et al., 2011	Mice (3xTg)	Entorhinal astroglial atrophy (1st month) precedes neurodegeneration (12 months of age).
	Kulijewicz-Nawrot et al., 2012	Mice (3xTg)	Medial prefrontal astroglial atrophy (1 month of age) precedes neurodegeneration (12 months of age).
AND	Rama Rao and Norenberg, 2014	Mice	Hepatic encephalopathy: selective astrocyte swelling.
	Hazell, 2009	Human Rats	Wernicke’s encephalopathy: loss of EAAT1 and EAAT2 astrocytic glutamate transporters.
ALS	Bruijn et al., 1997	Mice (SOD1^G85R^)	SOD1 astrocyte inclusions precede similar neuronal inclusions and later clinical signs.
	Rossi et al., 2008	Mice (SOD1^G93A^)	Abnormal spheroid-shaped astrocytes (75th day) in the ventral horns of the lumbar spinal cord, astrogliosis of the ventral and dorsal horns before clinical sings and evident neuron number reduction (100th day).
	Papadeas et al., 2011	Rats (SOD1^G93A^)	Selective astrocytic mutation of SOD1^G93A^ leads to ALS signs.
	Tong et al., 2013	Rats	Selective astrocytic mutation of Tar DNA-binding protein 43 (TDP-43) leads to ALS signs.
SCA	Custer et al., 2006	Mice	Bergman glia expressing ataxin-7 lead to SCA symptoms.
HD	Faideau et al., 2010	Mice Human	Selective expression of mutant Huntingtin in mice astrocytes leads to HD signs. Astrogliosis in the dorsal stratum in two human brain samples without clinical symptoms (grade 0).
	Bradford et al., 2009	Mice	Selective expression of mutant Huntingtin in mice astrocytes leads to HD signs.

*FTD, Frontotemporal dementia; PD, Parkinson disease; AD, Alzheimer’s disease; AND, Acquired neuropsychiatric disease; ALS, Amyotrophic lateral sclerosis; SCA, Spinocerebellar ataxia; HD, Huntington disease.*

Human anatomical material is scarce regarding this point because cadaveric material, such as brains and spinal cords, belongs mostly to patients who at the time of their death were already at late stages of their pathological process.

However, there are some observations in animal experimentation, which situates astrocytic damage at the beginning of the pathological timeline. Examples are as follows:

### Frontotemporal Dementia

In recent human studies astrocytes showing apoptotic signs were frequently found at the beginning of the disease when neuronal loss was yet very rare (Broe et al., 2004). Moreover, in a different study which focused on histological characteristics, astrogliosis was found to be present at early stages of the disease (Kersaitis et al., 2004). In later-stage cases, astrocytic apoptosis markers increased while astrogliosis was found with less frequency (Broe et al., 2004; Kersaitis et al., 2004). These findings support the notion that glial pathology occurs at the very starting of the illness besides being a major feature during the course of the disease.

### Parkinson Disease

In an extensive research done in human brains, Braak et al. (2003) demonstrated that α-synuclein deposition involves a wider brain area than the one that becomes symptomatic and that these symptoms appear at a later stage on the course of the disease, once neurons become affected at a high percentage. Curiously, the cells that do develop changes at early stages of intracellular α-synuclein deposition are astrocytes. In animal models, the accumulation of astrocytic cytoplasmatic α-synuclein eventually leads not only to astrocytic dysfunction but also to microglia activation through the release of cytokines and chemokines. These changes, in turn, determine the injury and eventually the non-cell autonomous killing of neurons (Halliday and Stevens, 2011).

In addition, several of the proteins codified by the altered genes in autosomic recessive forms of hereditary PD (Parkin, PINK-1, DJ-1), are mainly concentrated in astrocytes (McGeer and McGeer, 2008; Halliday and Stevens, 2011).

### Alzheimer Disease

In one of the only two transgenic animal models that mimic both the plaque deposition and the neurofibrillary tangles observed in humans (Oddo et al., 2003) it has been observed that the early-term and start of the mid-term stages of the illness are characterized by the presence of hippocampal astroglia atrophy (6 months of age). This period is followed by the appearance of Aβ plaques and neurodegeneration in association with the persistence of astrocyte atrophy as well as astrogliosis in the periplaque area (Olabarria et al., 2010). Moreover, in the same mouse model early astrocytic atrophy was also found in the entorhinal cortex at 1 month of age (Yeh et al., 2011) and in the prefrontal dorsolateral cortex since the 3rd month of age (Kulijewicz-Nawrot et al., 2012), in both cases without later periplaque astrogliosis and preceding neuronal dysfunction by even broader lapses.

### Acquired Neuropsychiatric Diseases

In several acquired neuropsychiatric disorders such as hepatic encephalopathy, Wernicke encephalopathy and heavy metals encephalopathy astrocytes are the primary affected cells (Rönnbäck and Hansson, 1992; Hazell, 2009; Rama Rao and Norenberg, 2014). Their dysfunction is believed to determine neuronal injury and eventually death through glutamate toxicity. Astrocytes are the only brain cells capable of metabolizing ammonia through the enzyme glutamine synthetase. However, the excessive concentration of ammonia in hepatic encephalopathy determines an uncommon availability of glutamate precursors as well as an increased production of reactive oxygen and nitrogen species which ultimately lead to astrocytic mitochondrial dysfunction (Rama Rao and Norenberg, 2014).

### Amyotrophic Lateral Sclerosis

Several observations support the idea that there is an early if not primary astrocytic damage in this disease (Sica, 2012).

To begin with, it was observed that transplantation of mutated SOD 1 expressing glial progenitors, able to differentiate into astrocytes, into the spinal cord of wild type rats induce motor neurons degeneration and clinical symptoms *in vivo* (Papadeas et al., 2011; Tong et al., 2013).

Furthermore, Rossi et al. (2008) employing the transgenic mouse model expressing the human SOD1^G93A^, identified a degenerative process involving a subset of astrocytes confined to the microenvironment of the spinal motor neurons. More importantly, the time-course of appearance of these cells was at the 75th day of age, that is, before the loss of motor cells. Moreover, the quoted astrocyte degeneration and its consequences were experimentally slowed down by the injection of 2-methyl-6-pyridine, a selective antagonist to the mGluR5 glutamate receptor. Therefore, glutamate toxicity seems to have a major role in astrocyte and neuron degeneration in mutant SOD1^G93A^ mice. These findings are consistent with the previously reported selective up-regulation of mGluR5 in astrocytes in the ventral spinal cord of ALS patients (Aronica et al., 2001).

Other notorious publication that has been referred to ever since is Bruijn et al. (1997) description of findings in genetically induced ALS mice. In this experimental study, transgenic and control mice were sacrificed at different ages and their CNS analyzed. They noted that the first pathological findings were astrocytic cytoplasmic aggregates that were approximately 1 month later observed in neurons and which preceded the clinical disease onset by 2.5 months.

### Spinocerebellar Ataxia (SCA)

Custer et al. (2006) demonstrated that in the polyglutamine disease SCA7, Purkinje cells undergo non-cell-autonomous degeneration in transgenic animals. They generated mice expressing ataxin-7 only in Bergmann glia (a specialized type of cerebellum’s astrocytes in close contact with Purkinje cells), which was sufficient to produce ataxia and neurodegeneration. In a similar fashion, Garden et al. (2002) also demonstrated that transgenic mice expressing ataxin-7 with 92 glutamines repeats (92Q) in non-neural cells at the cerebellum promoted Purkinje cells’ degeneration and therefore developed a dramatic neurological phenotype presenting as a gait ataxia and culminating in premature death despite the absence of its expression in Purkinje cells.

### Huntington’s Disease (HD)

In animal mouse models it was observed that mice expressing mutated Huntingtin (mhtt) in neurons show mild or no neurological sings, while mice expressing mhtt only in astrocytes have a reduced expression of astrocyte glutamate transporters and end up developing neurological deficits and an earlier neuronal death than when compared with control transgenic or wild-type mice (Gu et al., 2005; Bradford et al., 2009).

These observations are also supported by cultures results, where co-culturing wild mice neurons with astrocytes that over-express mhtt have shown that neurons undergo apoptosis, once more suggesting that those astrocytes have a deleterious effect onto normal neurons (Shin et al., 2005).

In humans, Faideau et al. (2010) observed reactive astrocytosis in the dorsal striatum in grade 0 HD subjects, when no neuronal damage is still apparent, with dorso-ventral striatum progression through grade 4 specimens. Despite being only two 0 HD human brain samples, we believe that these findings deserve at least further investigation.

## Conclusion

It has broadly been observed an early astrocytic dysfunction in the PDDs of the CNS. In the present mini review, this concept has been exemplified in CNS at its whole length: starting in the spine or the brain stem in ALS patients, ascending to the midbrain in PD, the cerebellum in SCA-7, the striatum in HD, the hippocampus in AD, the prefrontal cortex in FTD and, finally, compromising in a more subtle fashion the whole cortical gray matter in SCZ.

We advocate that these observations obtained from different degenerative pathologies, but mostly from experimental animal studies, may be the trees from a forest characterized by primary astrocytic dysfunction as the main process starting them.

We believe that in these particular pathologies, investigations conducted on human brain samples are absolutely necessary to confirm and expand the findings achieved in animal studies. This is based on the fact that particularly in cognitive pathology, anatomopathological findings do not necessary correlate with clinical symptoms (hence the actual disease) and that healthy animals do not acquire the high mental functions later to be lost, and therefore their analysis does not necessarily represent the human reality.

As a non yet explored approach, were astrocytic dysfunction to be of a diffuse fashion, the classically end-stage affected brain areas could be the ones to harbor early astrocytic pathology without neuron disease. Were astrocytic pathology to be restricted to a focused cerebral region, early changes in already symptomatic patients are unlikely to be studied although Parkinson and Alzheimer’s disease may be the exceptions due to their known sequence of progression.

We support the idea that a study with human samples focused in the context of the different diseases so far mentioned might allow to ascertain through detailed description characteristics previously overlooked, thus obtaining hints of a paradigmatic physiopathological process in which the astrocytes might be the main players.

## Author Contributions

All the listed authors have agreed on all of the contents and have contributed equally to its production.

## Conflict of Interest Statement

The authors declare that the research was conducted in the absence of any commercial or financial relationships that could be construed as a potential conflict of interest.
